# Progress towards Adjuvant Development: Focus on Antiviral Therapy

**DOI:** 10.3390/ijms24119225

**Published:** 2023-05-25

**Authors:** Annalaura Brai, Federica Poggialini, Claudia Pasqualini, Claudia Immacolata Trivisani, Chiara Vagaggini, Elena Dreassi

**Affiliations:** 1 Department of Biotechnologies, Chemistry and Pharmacy, University of Siena, Via Aldo Moro 2, I-53100 Siena, Italy; annalaura.brai@unisi.it (A.B.); federicapoggialini91@gmail.com (F.P.); pasqualini5@student.unisi.it (C.P.); chiara.vagaggini@student.unisi.it (C.V.); 2 Department of Pharmaceutical Sciences, University of Vienna, 1090 Vienna, Austria

**Keywords:** vaccines, small molecules, immune modulators, immune potentiators, formulations

## Abstract

In recent decades, vaccines have been extraordinary resources to prevent pathogen diffusion and cancer. Even if they can be formed by a single antigen, the addition of one or more adjuvants represents the key to enhance the response of the immune signal to the antigen, thus accelerating and increasing the duration and the potency of the protective effect. Their use is of particular importance for vulnerable populations, such as the elderly or immunocompromised people. Despite their importance, only in the last forty years has the search for novel adjuvants increased, with the discovery of novel classes of immune potentiators and immunomodulators. Due to the complexity of the cascades involved in immune signal activation, their mechanism of action remains poorly understood, even if significant discovery has been recently made thanks to recombinant technology and metabolomics. This review focuses on the classes of adjuvants under research, recent mechanism of action studies, as well as nanodelivery systems and novel classes of adjuvants that can be chemically manipulated to create novel small molecule adjuvants.

## 1. Introduction

Adjuvants—as indicated by the Latin etymology of the word (*adjuvare*, which means “to help”)—are defined as substances added to vaccines to boost the immune system’s response to the antigen and lengthen its duration. The use of adjuvants in vaccine development takes advantage of the many benefits these substances can offer, such as reducing the amount of antigen required for each vaccination dose and the frequency of booster vaccinations or improving the stability of the antigen component by lengthening its half-life and, consequently, enhancing its immunogenicity [1]. Adjuvants can be classified based on their mechanism of action, chemical properties, or based on their origin (synthetic, natural, endogenous) [2]. The adjuvants superfamily comprehends many different substances, in particular small or macromolecules capable of activating or potentiating immune signaling or delivery systems [3,4,5]. Immune potentiators are compounds capable of activating immune signal in adults or vulnerable populations; among them are agonists of pattern recognition receptors (PRRs), such as RIG-I like receptors (RLR) [6], stimulators of interferon genes (STING) [7], Toll-like receptors (TLR) [8,9,10,11,12,13], and NOD-like receptors (NLRs).

Delivery systems are adjuvants capable of ameliorating and extending vaccine protection, such as emulsions and nanoformulations, similarly to liposomes, virus-like particles, and virosomes [3,4,5]. According to the desired type of immune response, antigens should be properly formulated with the opportune adjuvant or adjuvants combination, to obtain the best possible response with the fewest side effects [6]. Proper formulations have been developed so far by combining different families of adjuvants, in particular alum with liposomes or emulsions [7]. Identifying the proper adjuvant combination can be extremely important, and many clinical studies are actually ongoing to investigate their efficacy in different pathologies, in particular cancer [8]. Because adjuvants’ applications range from pathogens to allergies, autoimmune disorders and cancer, the key mechanism needs to be properly understood in order to target only specific pathways avoiding potential toxicity.

Even if vaccines’ trials follow strictly regulated guidelines, many concerns about their safety have arisen over the years [9], in particular during COVID-19 vaccination campaigns [10]. The potential toxicity of vaccines is sometimes attributed to the adjuvants contained. Several concerns also emerged about the potential toxicity of the most characterized and safer adjuvants, such as alum derivatives. Even if alum content in licensed adjuvants ranges from 0.8 to 0.125 mg per dose, concerns about neurotoxicity and autism emerged in recent years [11]. In deep contrast, other studies demonstrate that aluminum neurotoxicity proceeds after chronic administration, and regulatory organs limited the Tolerable Weekly Intake (TWI) of aluminum in foods to 1 mg per kg of body weight [12]. It is interesting to note that sometimes adjuvants are constituted by lipids, as in the case the nanoformulated liposomes, or other endogenous risk-free macromolecules. EMA and FDA have approved 47 vaccines, but most of the adjuvants included in these preparations are members of the oldest classes of alum adjuvants, or liposome formulations [1]. This choice is probably due to the known tolerability of these classes of adjuvants, and the costs related to the search for novel compounds. In fact, different classes of small molecule immunopotentiators have been discovered in recent years; nevertheless, as for drugs, these compounds need proper and time-consuming preclinical and clinical trials to assess their efficacy and safety. Among the most recent and interesting classes, several PRRs agonists have been reported with promising results for the adult population. Recent studies highlighted the efficacy of small molecules capable of activating immune responses via mitochondrial stress pathways, thus overcoming PRRs pathways [13]. This review focuses on the mechanisms of action, on the developed adjuvants’ classes, with particular emphasis on the novel pathways that can be targeted to create novel adjuvants.

## 2. Mechanism of Adjuvanticity

Although adjuvants are commonly used in the formulation of billion-dose vaccines, the mechanisms of action are still poorly understood. Thus, a deep understanding of the way of action and the immunological mechanisms involved in the immune system response to pathogens represents a crucial step for the development of new adjuvants. Recently, significant attention has been paid to a deeper understanding of how vaccination adjuvants stimulate the immune response. Thanks to the recent advances in immunological research, it has been possible to elucidate some of the mechanisms by which adjuvants act, such as the depot effect and the release of cytokines and chemokines, the mobilization of immune cells at the injection site, the induction of adaptive immune responses, the increase in the antigen immunogenicity, and the activation of antigen-presenting cells (APCs) [14,15]. Clarifying all the mechanisms by which adjuvants explicate their action will furnish crucial information on how adaptive immunity is promoted by the innate one, and help in the development of new potent vaccines. Adjuvants can be classified using a broad range of factors, including their physicochemical characteristics, origins, and modes of action; one of the most popular classification schemes divides vaccine adjuvants into two main groups, delivery systems and immunostimulators. Another class of adjuvants is represented by the mucosal ones which can act both as delivery vehicles or immunostimulatory compounds, such as chitosan and its derivatives (N-trimethyl and mono-N-carboxymethyl chitosan), cholera toxin (CT), and the heat-labile enterotoxins (LTK3 and LTR72). Novel delivery system adjuvants are reported in Table 1. Traditionally, delivery vehicles operate only as a depot for immunostimulatory adjuvants to activate cells of the innate immune system cells. Since there is now evidence that some delivery mechanisms can activate innate immunity, this classification is no longer accurate [16].

In fact, delivery vehicle adjuvants both work as antigen carriers and cause a local pro-inflammatory response by activating the innate immune system, resulting in the recruitment of immune cells to the injection site. The antigen-adjuvant complex induces the activation of pattern recognition receptor (PRR) pathways by acting as pathogen-associated molecular patterns (PAMPs). These phenomena lead to the induction of innate immune cells, resulting in the release of cytokines and chemokines, the same mode of action exploited by immune potentiators adjuvants [1,17,18,19]. Immunoadjuvants (Table 1) are immune potentiator compounds that enhance antibody production by direct stimulation of the innate immune system. Moreover, adjuvants acting as immunomodulators can stimulate the production of specific types of cytokines, thereby boosting the response of the immune system. For example, alum, Freund’s adjuvant, and CpG oligodeoxynucleotides have been reported to induce the production and release of some cytokines involved in the regulation of innate and adaptive immunity, such as interferons (IFNs), interferon-γ (IFN-γ), and interleukins (IL2 and IL12) [2]. Several cytokines have been reported to act as immune potentiators adjuvants stimulating antigen-specific serum/mucosal antibody and cell-mediated immunity. Among this family of substances, the most well-known cytokines adjuvants are granulocyte/macrophage colony-stimulating factor (GM-CSF), IFN, chemokines, and a few interleukins (IL-1, IL-2, IL-12-IL-15, IL-18) [15]. Moreover, the immunostimulatory adjuvants are useful for the recruitment of immune cells, such as macrophages, neutrophils, and dendritic cells (DCs); the activation of the APCs; and the prolonged accumulation of the vaccine in the site of injection. Recent studies have linked Toll-like receptors (TLR) to autoimmune systems, discovering the mechanisms by which TLR activate the innate immunity system that results in adaptive immunity and inflammatory response induction, ensuring long-lasting protection [20].

**Figure 1:**
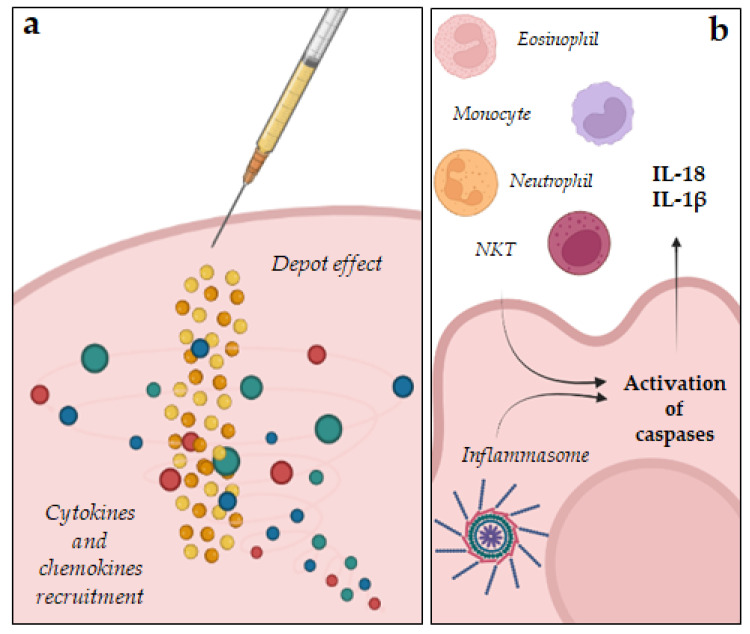
(**a**) Depot effect and cytokine and chemokines recruitment. (**b**) Immune cells recruitment and inflammasome activation.

Adjuvants can act as delivery systems, entrapping, adsorbing, or aggregating antigens and slowly releasing them over time. This mechanism, defined depot effect (Figure 1a), occurs at the injection site where adjuvants prevent the removal of the antigen due to hepatic clearance; this enhances the vaccine’s half-life and ensures a continuous stimulation of the immune system resulting in high antibody titers. Over the years, many examples of adjuvants acting through the depot effect have been described, such as liposomes, emulsions (both *o*/*w* and *w*/*o*), virosomes, and lipid or polymeric nanoparticles (NPs). Some of them have been developed to simulate pathogen membranes to transport, preserve, and release the antigens, and simultaneously enhance their immunogenic functions. Several types of liposomes, such as the traditional ones, the multilamellar vesicles (ICMVs), or the solid core liposomes, exploit their action also by promoting the depot effect.

**Figure 2:**
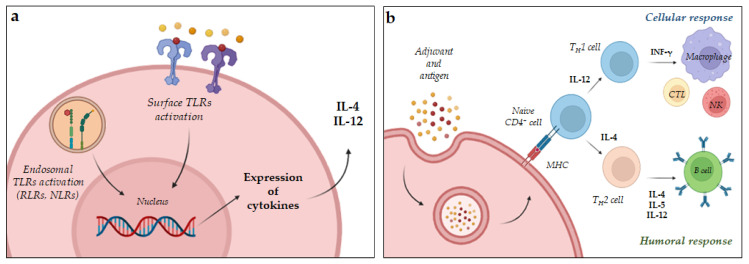
(**a**) Surface and endosomal TLR activation. (**b**) Enhancement of antigen presentation.

Water in oil emulsions, such as the complete Freunds adjuvant (CFA), and some NPs also act through the depot effect that ensures long-lasting immune responses [2,16]. Particulate adjuvants can induce immune responses by exploiting several mechanisms, such as up-regulating the release of cytokines and chemokines, inducing an inflammatory state at the injection site that activate the inflammatory cascade and recruiting innate immune cells. For example, the oil in water (*o*/*w*) emulsions MF59 and AS03 stimulate the recruitment of immune cells (neutrophils, monocytes, macrophages, and DCs) that transport both the antigen and the adjuvant to closer lymph nodes. The recruitment of immune cells in the injection site induces the activation of caspases, resulting in a further release of chemokines (IL-18, IL-33, IL-1β) which attract other DCs and prolong this phenomenon (Figure 1b). Furthermore, MF59 and AS03 increased at the site of injection the expression of CCR2, leukocyte-recruiting chemokines (e.g., CCL2, CCL3, and CCL5), as well as colony-stimulating factor 3 (CSF3). Similarly, alum induces a local pro-inflammatory microenvironment after injection that provokes the activation of the complement cascade leading to the recruitment of immune cells from the bloodstream [2,16]. Inflammasomes represent an important component of the innate immune system. They are required for an effective immune response to pathogens. When an inflammasome is activated, the cell secretes pro-inflammatory cytokines, such as IL-18, IL-33, and IL-1β, which boost the adaptive immune response (Figure 1b). Inflammasomes are cytosolic protein signaling pathways made of working components, such as a leucine-rich repeat (LRR) C-terminal or DNA-binding domain (HIN200), a caspase-1 effector and an adaptor protein ASC which activate inflammatory caspases. Granulocytes, T- and B-cells, monocytes, hepatocytes, neurons, microglia, and Langerhans cells all express inflammasomes that are responsible for recognizing pathogens and initiating an innate immune response. When an inflammasome is activated, it proteolytically cleaves pro-caspase 1, liberating the active form which converts pro-IL-1β and pro-IL-18 into the active species. Released from cells, ILs initiate inflammation and induce the immune response that protects against pathogens. Furthermore, IL-18 activates lymphocytes and stimulates the proliferation of T-cells and B-cells, the activity of natural killers (NKs), and the secretion of IFN-γ, TNF, IL-1, and IL-2. Thus, adjuvants acting as inflammasome activators represent successful strategies to enhance and sustain immune response strength. These adjuvants activate inflammasomes through similar mechanisms, including degradation of lysosomes, cathepsin release, and the formation of reactive oxygen species (ROS). Among the inflammasome activators, adjuvants, such as aluminum salts, chitosan, saponins, flagellin, and synthetic cation polymers can be found. Aluminum salts provoke lysosome damages which induce the production of cathepsin B involved in the formation of the inflammasomes, in particular the NOD-like receptor protein 3 (NLRP3); the active inflammasome triggers caspase-1 and stimulates the release of cytokines. Chitosan and nanoparticles (NPs) made of synthetic cation polymers activate the NLRP3 inflammasome and enhance the secretion of several interleukins (IL-2, IL-4, IL-6, IL-10, IL-17A, and TNF), IFN, and IgG titers, boosting both cellular and the humoral immune responses [21,22]. Adjuvants can boost the immune reaction to vaccines through a wide range of mechanisms, such as depot effect and the stimulation of innate immunity. The first line of defense against pathogens is represented by the innate immunity. In fact, early recognition of pathogens is a key step in developing adaptive immune responses. Adjuvants can induce innate immunity by activating cellular pattern recognition receptors (PRRs), which recognize PAMPs and damage-associated molecular patterns (DAMPs) and stimulate APCs. Due to the central role in the innate immune system, PRRS represents a strategic target for new adjuvants. Within the PRRs superfamily, TLR, distinguished into surface and endosomal receptors, are promising adjuvant targets because they can induce signaling pathways, resulting in the induction of key transcription factors, such as nuclear factor-B (NF-B). Adjuvants can also be used to target endosomal PRRs, such as nucleotide-binding oligomerization domain-like receptors (NLRs) and retinoic acid-inducible gene-I-like receptors (RLRs) (Figure 2a). The localization is strictly related to their properties; in fact, plasmatic TLR recognize pathogenic proteins and lipids, while endosomal ones are activated by nucleic acids. TLR induce NF-kB through the MyD88 pathway, resulting in the release of pro-inflammatory cytokines. TLR-based adjuvants replicate PAMPs produced during the infection and can, thus, be extremely effective against pathogens or diseases that normally induce PRRs. Despite the excellent immunostimulatory efficacy of PRR agonists, their use as vaccine adjuvants has limitations due to high manufacturing costs which represent a limit for future clinical applications [16,23,24,25]. APCs, such as dendritic cells (DCs), express a variety of PRRs that allow them to recognize several pathogenic constituents. When PRRs are activated by PAMPs, they initiate complex signal cascades that result in the production of cytokines and chemokines, which include interferons (IFNs), the enhancement of antigen presentation capacity, and the migration of DCs to lymphoid tissues, where they interact with T cells and B lymphocytes to initiate and shape the adaptive immune response. Matured DCs can also stimulate naive CD4+ T cells to differentiate into different T helper (Th) subsets (e.g., Th1 and Th2 cells), which help B cells produce antibodies. Several cytokines regulate Th cell differentiation; for example, cytokines such as IL-12, IL-15, and IL-27 regulate the development of naive CD4+ T lymphocytes in Th1 cells. In summary, Th1 cells predominate in response to intracellular pathogens, such as viruses and some bacteria, whereas Th2 cells predominate in response to large extracellular parasites [23]. DCs are also able to stimulate naïve cytotoxic CD8+ T cells into activated CD8+ T cells [26]. This phenomenon called “cross presentation” is necessary for inducing strong and durable cellular immunity against exogenous antigens, and for the effective prevention of viral diseases and cancer. It is still unclear how exogenous antigens are processed in DCs and presented to CD8+ T lymphocytes on MHCI; however, two different mechanisms have been proposed [27]. In the cytosolic pathway, antigens enter into the cytosol through endosomal vesicles, and are degraded by proteasome. In the vacuolar pathway, antigens are degraded in lysosomal compartments, independently from proteasome activity. Aluminum, saponins, and TLR adjuvants can act using this mechanism.

Antigen presentation elicited by the major histocompatibility complex (MHC) on APCs, represents a critical step in the activation of adaptive immunity. Many adjuvants, such as alum, emulsions, and NPs, were supposed to function by “targeting” antigens to APCs, enhancing the antigen presentation by MHC. To date, it has not been clarified yet whether the mechanism through which adjuvants increase antigen presentation contributes to the development of the adaptive immune system. For instance, alum has been shown to boost the antigen uptake by DCs, as well as prolong the duration of antigen presentation. Antigen size appears to be important in modulating antigen presentation efficiency. Large lipid vesicles are found in early endosomes/phagosomes, where they increase antigen presentation, whereas smaller vesicles are found in late lysosomes, where they decrease antigen presentation [16].

## 3. Types of Adjuvants

### 3.1. Aluminum Salts

Aluminum-based adjuvants (ABA) have been first discovered in 1926 [28] and are currently the most commonly used adjuvants in vaccines worldwide [29]. The first aluminum adjuvant employed was aluminum potassium sulphate, commonly referred to as “alum”, prepared by direct precipitation of a solution of the antigen and the adjuvant with a base (alum-precipitated vaccines). Today the antigens are adsorbed onto a preformed gel of aluminum salt (direct adsorption), offering more advantages in terms of standardization and reproducibility of commercial preparations [30]. Currently, the traditionally used alum has been almost totally replaced by boehmite-like aluminum oxyhydroxide (Alhydrogel^®^, Croda, Frederikssund, Denmark) and amorphous aluminum hydroxyphosphate (AdjuPhos^®^, Croda, Frederikssund, Denmark) [31,32]. Two novel adjuvants are the sulphate salt of aluminum hydroxyphosphate (AAHS), currently used in certain formulations of Human Papilloma Virus (HPV) vaccine [33,34] and Imject^®^Alum (Pierce, Rockford, US), composed of amorphous aluminum hydroxycarbonate and crystalline magnesium hydroxide [35]. In human vaccinations, ABA have been primarily used in vaccines against tetanus, diphtheria, pertussis, poliomyelitis, hepatitis A and B, and human papillomavirus (HPV). A list of FDA-licensed human vaccines containing ABA is reported in Table 2. ABA are widely employed also in veterinary vaccines [36,37] against bacterial [38,39], viral [40,41], and parasite infections [31,42].

Despite the long history of use, light has still to be cast on the mechanisms behind the immunostimulating properties of ABA [30].

Initially, the adjuvant properties of ABA were first ascribed to a “depot effect” [44]. According to this hypothesis, the antigen particles are slowly released in the body from the insoluble salt particles over a long period of time, allowing a prolonged exposure of the antigen to the immune system and a potentiated immunostimulation, resulting in a higher antibody titer [45]. However, recent findings challenged this theory, demonstrating that the antigen retention at the inoculation site was not required for the resulting immune response, but it was the magnitude of the inflammation at the inoculation site to account for the adjuvant effects. Thus, apart from acting as a gradual-release system, ABA have other major effects on immunostimulation, such as innate immunity cells recruitment and activation, inflammatory mediators release and adaptive immunity stimulation via Th_2_ cells induction [46].

Upon administration, innate inflammatory cells, such as neutrophils, eosinophils, dendritic cells (DC), and monocytes, are recruited to the site of injection [30]. Despite the fact that the activity of many immune adjuvants is based on Toll-like receptors (TLR) signaling, aluminum salts apparently do not elicit a TLR-based response [46,47,48].

Similarly to uric acid, released in the cytoplasm after cell damage as insoluble monosodium urate (MSU) crystals, it has been shown that the particulate nature of ABA facilitates phagocytosis by macrophages and antigen uptake in APCs [44]. Furthermore, the cytotoxicity of ABA induces secretion of heat-shock proteins (such as *hsp*70) and other DAMPs, such as uric acid in the form of MSU [43].

**Figure 3:**
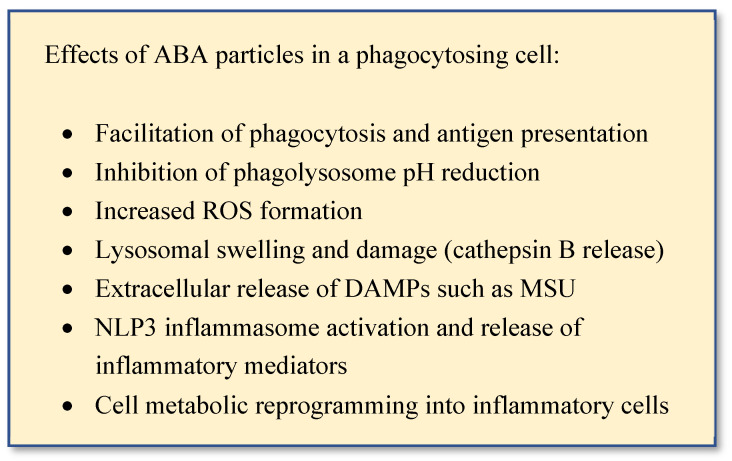
Effects of aluminum-based adjuvants (ABA).

NLR family-pyrin containing domain 3 (NLP3) is a member of the nucleotide-binding oligomerization domain (NOD)-like receptors family (NLR) and is a cytoplasmic pattern recognition receptor (PRR) with a crucial role in the regulation of innate immune signals [49]. NLP3 is activated by potassium efflux, thereby acting as a sensor of membrane integrity [50]. When triggered by stimuli such as ATP, asbestos, silica, aluminum adjuvants, or MSU, NLP3 associates with adapter protein apoptosis-associated speck-like protein containing a CARD (ASC) and inactive pro-caspase-1 to form the NLP3 inflammasome multimeric complex [46]. The autoproteolytic cleavage of procaspase-1 into active caspase-1 cleaves the proinflammatory cytokines precursors pro-IL-1β, pro-IL-18, and pro-IL-33 into their active and secreted forms [44,51]. It has been hypothesized that aluminum salts may activate NLP3 inflammasome either directly, through phagolysosomal damage and subsequent cathepsin B release [52] after phagocytosis, or indirectly through the release of MSU [53]. In addition to NLP3-mediated inflammation, the polarization of sentinel cells into active macrophages and APCs, increased the production of phagosome reactive oxygen species (ROS), phagosome acidification disturbance and cell metabolic reprogramming via hypoxia inducible transcription factor-1α (HIF-1α) are other recently disclosed mechanism contributing to the immune stimulating properties of ABA [30] (Figure 3).

Activation of sentinel cells into APCs is crucial for an adaptive response, thereby linking innate immunity with adaptive immunity. ABA increases antigen presentation on activated DCs via major histocompatibility complex class II (MHCII) molecules. MHCII-antigen presented sites engage CD4+ T cells, which differentiate and activate B cells, which, in turn, produce mainly IgG, driving humoral immunity [54]. Aluminum salts boost preferentially an antibody-mediated immune response, through T_FH_ cells and IL-4 signaling [55], resulting in the production of IgG1 and induce differentiation of Th_2_ cells that drive eosinophilic inflammatory response through IgE. In contrast to the strong Th2 responses, alum is less efficient against infections that need Th1-cell mediated protection. In mice studies, it has been demonstrated that alum indirectly inhibits Th1 responses due to IL-4 activation [56]. Lymphokines produced through Th1 response are fundamental inductors of complement fixing IgG2a antibodies, thus macrophages stimulators [57]. Additionally, cells of the native immune system can develop adaptive properties (“trained immunity” [58]) and aluminum adjuvants could be implied in the induction of trained immunity [30]. The role of NLP3 inflammasome and caspase-1 in antibody response induction, however, is still controversial [59].

After almost a century of use, aluminum salts are still a milestone in vaccine adjuvants because of their well-established record of safety and efficacy [60]. ABA are very well tolerated and only some minor local reactions have been reported [30,31], such as injection site pain, swellings, erythemas, and rarely granulomas and allergic reactions, which reflect their mode of action through inflammasome activation, proinflammatory mediators, accumulation of phagocytic cells, and antibody production [61]. Rare cases of contact dermatitis in some immunized subjects, post-injection headaches, arthralgia and myalgia, and persistent swelling have also been described [62]. A great debate has been raised concerning the long-term toxicity of aluminum adjuvants, including effects such as Alzheimer’s Disease (AD), chronic autoimmunity, and multiple sclerosis [29]. The Autoimmune Syndrome Induced by Adjuvants (ASIA) [30] indicates a group of adverse effects comprising Gulf war syndrome, macrophagic myofasciitis, siliconosis, and post-vaccination phenomena related to adjuvant exposure [63]. Furthermore, since aluminum ions have been implied in the pathogenesis of AD [64,65], concerns have been raised regarding the biopersistence and potential neurotoxicity of ABA, but this correlation has never been demonstrated [29]. Despite further knowledge being needed to update and confirm the safety profile of ABA, the risk–benefit profile of aluminum salts as adjuvant remains extremely positive [61] and confirms them as the gold standard of vaccine adjuvants.

### 3.2. STING Agonists Adjuvants

Cyclic GMP-AMP synthase/stimulator of interferon genes (cGAS/STING) pathway is part of a network of cytosolic PRRs of the innate immune system, which monitors the cell cytoplasm to sense danger stimuli [66]. The stimulation of the pathway activates downstream nuclear factor-κB (NF-κB) and interferon regulatory factor 3 (IRF3), increasing the transcription of type I interferons (IFN-1) and other proinflammatory cytokines, thereby boosting antigen presentation and immune response [67]. The key role of cGAS/STING in immunoregulation [68] distinguishes it as an important target for immunotherapies, especially cancer-related, and is the rationale behind the use of STING agonists as promising vaccine adjuvants [2].

**Figure 4:**
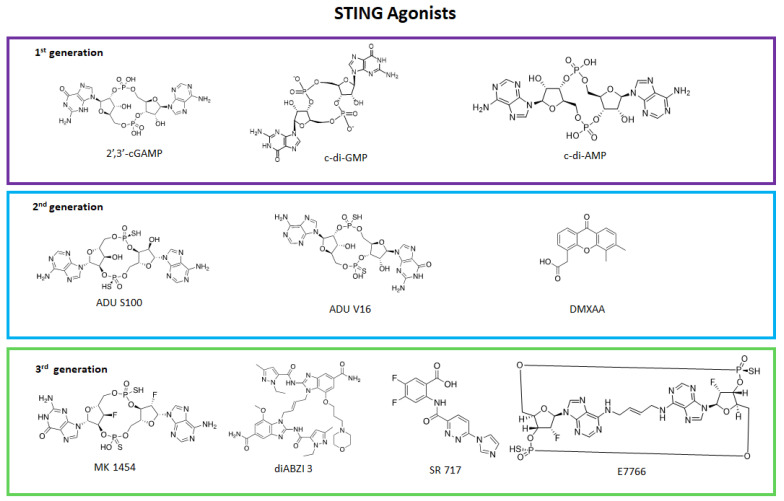
Two- dimensional structures of small molecule STING agonists.

STING agonists (Figure 4) mostly find application in oncology and virology, and are represented by cyclic dinucleotides (CDNs), non-nucleoside small molecules (NCDNs), cytosolic double-stranded DNA (dsDNA), manganese ion, ionizable lipids, and polymers [67].

Cyclic dinucleotides such as 2′,3-cyclic guanosine monophosphate—adenosine monophosphate (cGAMP), cyclic dimeric guanosine monophosphate (c-di-GMP), or cyclic dimeric adenosine monophosphate (c-di-AMP) are natural agonists of the pathway, but their poor pharmacokinetic (PK) properties as high polarity and short half-life due to ectonucleotide pyrophosphatase/phosphodiesterase (ENPP1) enzymatic degradation, strongly limit their use [69,70]. To improve the poor PK profile of cyclic dinucleotides, phosphorotioate analogues, such as ADU-S100, have been tested [71,72,73] and ENPP1 inhibitors [74] also represent a novel promising strategy [75]. To select candidates with better activity, the search for rigid analogues that mimic the binding conformation of CDNs and STING led to a new class of agonists, the macrocycle-bridged stimulators (MBS), such as E7766 [76]. To overcome the PK limitations of cyclic dinucleotides, research focused also on NCDNs, such as the xantones 5,6-dimethylxanthenone-4-acetic acid (DMXAA) [77] and α-mangostin [78], and dimeric amidobenzimidazole (diABZI) derivatives, which gave promising result for immunotherapy in oncology and against severe acute respiratory syndrome coronavirus 2 (SARS-CoV-2) [71,79,80,81].

Strategies that induce the release of cytosolic dsDNA have also been employed as a way to trigger the STING pathway [82], such as the use of radiotherapy [83], alum-based adjuvants [84], and chemotherapeutics, such as cisplatin or doxorubicin [85,86]. Inorganic manganese [87] was found to activate cGAS and act as an indirect agonist of STING by enhancing the production of the second messenger cGAMP [88]. Among other agonist classes, we enumerate polymers, such as chitosan [89] or PC7A [90,91], and nanoparticle-based ionizable lipids [92,93,94,95]. A summary of STING agonists currently in clinical trials is reported in Table 3.

The trigger for cGAS/STING signaling is cytosolic dsDNA, an important hallmark of cellular damage. As a result, cGAS, acting as a direct DNA receptor, catalyzes the transformation of dsDNA into the second messenger cGAMP, which induces activation and oligomerization of STING [96]. STING oligomer activates TANK-binding kinase 1 (TBK1), which recruits IRF3 and induces transcription of type I IFN-stimulated genes through NF-κB [97]. Downstream induction of autophagy and NLP3 inflammasome activation increases pathogen clearance and is strongly implied in autoimmune and inflammatory diseases, ageing, and tumor-associated inflammation [98,99]. STING agonists are generally related to cyclic dinucleotides, such as cGAMP, c-di-GMP, and c-di-AMP, found as metabolites of various micro-organisms [100].

Agonists of the STING pathway directly induce maturation and upregulation of MHCII molecules of DCs, increase antigen presentation, and T cells priming and indirectly contribute to the previous effects through inflammatory cytokines. STING agonists also enhance adaptive immunity responses, boosting humoral immunity via IgG1 and IgG2 production, spleen germinal center induction, and memory B cells stimulation [67]. Furthermore, type I IFN induces differentiation of CD4 T cells into Th_1_ and T_FH_ cells, significantly helping the priming of B cells, and also promotes CD8 T cells activation and proliferation, important for tackling resistant tumor cells [101,102]. The multiple immune responses orchestrated by the stimulation of STING make this pathway an attractive target for immune therapies. Despite acute activation of cGAS/STING provides undoubted benefits against pathogens and cancer cells, a chronic activation may result in an IFN-driven systemic inflammation, inducing a cytokine storm [103] (cytokine release syndrome [104]), similarly to sepsis. In addition to the potential of inducing a systemic inflammatory reaction, the other major issues observed in response to STING overstimulation are a lack of cell and tissue specificity and lymphocyte toxicity [105,106,107]. These premises, coupled with the challenging PK properties, complicate the scenario of a systemic administration of STING agonists, which are generally administered intratumorally [66]. The employment of drug-carrier technologies, such as nanoparticles, lipid-based carriers, and antibodies is crucial [108] to achieve more selective targeting, combined with improved delivery and efficacy. Despite their challenging PKs and narrow therapeutic index for systemic use, STING agonists represent a very promising adjuvant class, and an optimization of their formulation is needed to further improve their adjuvanticity.

### 3.3. TLR Ligands

#### 3.3.1. Toll-like Receptors

Most of the vaccines on the market consist mainly of a single adjuvant but often the protective immune response is not up to the mark for effective use of vaccines. Therefore, significant Toll-like receptors (TLR) are a key component of innate immunity, providing defensive inflammatory responses to invading pathogens. Human TLR includes 10 members (TLR1-10) that can span through the membrane of the cell surfaces (TLR1, 2, 4, 5, 6, 10) or can be localized on the endoplasmic reticulum membranes (TLR3, 7, 8, 9) and that are involved in the recognition of different PAMPs (Table 4) [109,110].

The binding of the PAMPs induces TLR homodimerization (TLR3, 4, 5, 7, 8, 9) or heterodimerization (TLR1/2 or TLR2/6) that brings together the two toll/IL-1 receptor (TIR) domains, allowing the adaptor protein MyD88 (myeloid differentiation primary response-88) to bind the complex [111]. TLR3 are the unique receptors that bind a different adaptor, the TIR-domain-containing adapter-inducing interferon-β (TRIF) protein [112]. Differently from the other TLRs, TLR4 signaling can take place via two separate pathways which involved the MyD88 signal adaptor protein or TRIF [113]. Once activated, the TLR led to increased immune cell trafficking and an adaptive immune response.

**Figure 5:**
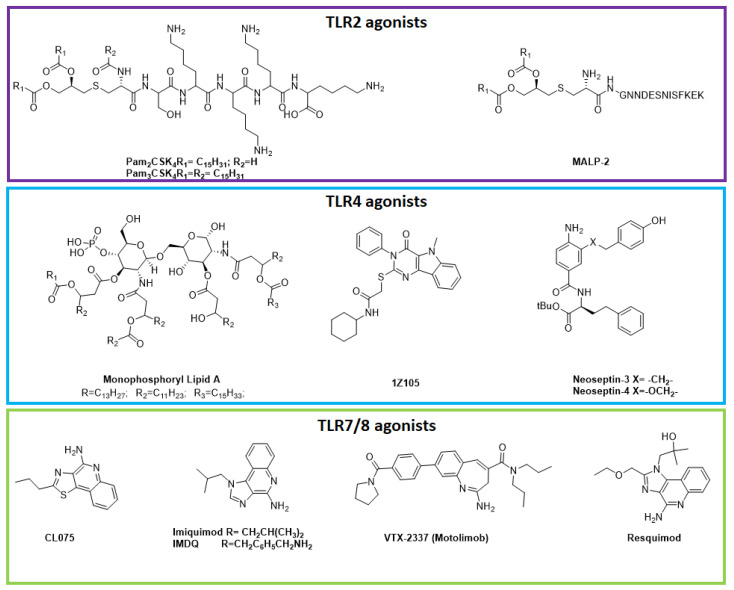
2D structures of small molecule Toll−like receptors agonists. TLR2 agonists (violet upper panel), TLR4 agonists (cyan, middle panel), TLR 7/8 agonists (green, bottom panel).

Since TLRs act as immune potentiators, their agonists can be used as vaccine adjuvants. The type of immune response generated by vaccines depends on the signaling pathway activated by the specific TLR and their adaptor protein. While the majority of TLR pathways lead to Th1 immune responses, TLR2 induces a Th0, Th1, or Th2 responses, and TLR3 activates the NF-kB pathway [55]. There are several TLR agonists used in vaccine formulation that are summarized in Table 1, while structures of TLR agonists are reported in Figure 5 and TLR agonists in clinical trials are in Table 5.

#### 3.3.2. TLR2 Agonists

TLR2 can recognize a great variety of PAMPs since they can heterodimerize with TLR1 and TLR6. L-pampo is a potent adjuvant system constituted by a complex formed by PAM_3_CSK_4_ (PAM_3_), TLR1/2, a poly(I:C), and TLR3 [114]. It is used in the hepatitis B virus (HBV) vaccine, it induces a cell-mediated immune response, increasing the CD4+ T cells levels [115]. Currently, it is under investigation as an adjuvant in SARS-CoV-2 vaccines that uses RBD (receptor binding domain), S1 antigen and RBD-Fc as viral antigens, which is demonstrated to evoke strong humoral and cellular immune responses [114].

TLR2 recognizes the bacterial lipoproteins, and, for this reason, synthetic lipopeptides derived by bacterial LPS were developed as vaccine adjuvants. MALP2 (macrophage activating lipopeptide 2, Figure 5) is derived from *Micoplasma fermentans* and uses the TLR2-MyD88 signaling pathway to activate immune cells [116]. PAM_2_CSK_4_ and PAM_3_CSK_4_ are two TLR2 agonists evaluated as adjuvants in vaccines against leishmania [117], malaria, and influenza [118]. Due to their size, chemical complexity, and hydrophobicity, TLR2 agonists are not often used in vaccine development [119], even if the research on automatic peptide synthesis in the solid phase can aid the discovery of novel more simple derivatives.

#### 3.3.3. TLR3 Agonists

TLR3 are endosomal receptors that detect viral dsRNA [120]. Poly(I:C) is an adjuvant that structurally resembles the viral RNA. It is able to induce the IFN-I and IFN-III production and stimulate the Th1 cytokine response [121]. The activation of the MVS pathway (RIG-1 and/or MDA5) causes human toxicity, for this reason novel poly(I:C) derivatives, poly(ICLC) [122] and poly(IC12U), have been developed [123].

Poly(ICLC) is a poly-L-lysine in carboxymethyl cellulose that stimulates the IFN production [122]. It induces the expression of the inflammasome and the complement system and is used in vaccine candidates against *Plasmodium falciparum*, [124] HIV [125] and cancer [126], demonstrating a strong ability to elicit the Th1 response. Due to the high immunostimulatory effect and high resistance to serum nuclease of this adjuvant, poly(IC12U) was designed. This new substance shows a mismatch of the uracil and guanosine residues that led to a lower toxicity (it does not bind MDA5), and a lower production of IFN-I [123].

#### 3.3.4. TLR4 Agonists

TLR4 recognizes the bacteria lipopolysaccharides through MD-2, the co-receptor myeloid differentiation factor-2, and CD14 [127]. The immunostimulatory activity of LPS is due to the lipid A region, and the variation of the fatty acyl chains reflects a different biological activity [128]. Eritoran is a TLR4-MD2 agonist. It is a synthetic derivative of lipid A characterized by four lipid chains, one of these containing a double bond in *cis* configuration [129]. The 3-O-desacyl-4′-monophosphoryl lipid A (MPLA, Figure 5) [130] and glucopyranosyl lipid A (GLA) [131] show low pyrogenicity and strong immunopotentiator characteristics. LeIF (Leishmania eukaryotic initiator factor) [132] and neoseptins (synthetic peptidomimetic compound, Figure 5) [133] are two non-glycolipid ligands that do not resemble LPS but are able to activate TLR4 as the natural ligand does. High-throughput screening for small compounds that activate the NF-B pathway in THP-1 cells resulted in the discovery of a small-molecule TLR4 specific agonist that belongs to the class of pyrimidoindoles (1Z105, Figure 5). 1Z105 was determined to be a safe TLR4 agonist, and other studies are actually ongoing. TLR4 can activate a robust TRIF-mediated cellular response characterized by the presence of polyfunctional CD8+/CD4+ T cells and enhanced CTL activity against both cancers and infectious diseases [119].

#### 3.3.5. TLR5 Agonists

TLR5 are expressed by several immune cells and are involved in bacterial flagellin recognition. The binding of a ligand to TLR5 induces the activation of the inflammation pathway, with the release of inflammatory mediators, including TNFα, IL-1β, IL-6, and nitric oxide, evoking Th1 and Th2 responses [134]. When administered with an antigen, flagellin induces a mucosal immune response that is essential in protecting against respiratory and gastrointestinal infections [135]. Flagellin from *Salmonella typhimurium* has been formulated with PR8 influenza virus (IPR8), HA (H5N1) or Avian influenza virus (AIV) H5N1 antigens, where it was demonstrated to elicit a strong immune response, with IgA production [1]. Modification to flagellin led to chimeric flagellins or complexes of flagellin antigens in live attenuated bacteria, such as *Mycobacterium tuberculosis*, *Vibrio cholerae*, *Streptococcus pyogenes*, *Listeria monocytogenes*, *Enterotoxigenic Escherichia coli* (ETEC), and used in animal models [136]. Three vaccines that use flagellin as adjuvant are in the clinical trial phase, two against influenza virus [137,138] and one against *Yersinia pestis* [139].

#### 3.3.6. TLR7/8 Agonists

TLR7/8 induce a Th1 immune response and produce high levels of type I IFN, IL-12, TNF-α, and IL-1β [140]. TLR7/8 and TLR9 agonists are able to activate and promote the clonal expansion of cDCs and plasmacytoid dendritic cells (pDCs) mobilizing CD14^+^ CD16^+^ inflammatory monocytes and CD14dimCD16^+^ patrolling monocytes [141].

Imiquimod (R837, Figure 5) is currently approved and licensed for the treatment of genital warts [142], superficial basal cell carcinoma [143], and actinic keratosis [144]. Another isoquinolin derivative, resiquimod (R848) has antiviral and anticancer therapeutic uses and is under evaluation for the melanoma treatment [145]. Different structurally related oxoadenine compounds have been developed, even if other preclinical studies are necessary to demonstrate their efficacy [146]. CL075 is a structurally related heterocyclic compound with a fused quinoline-fused thiazole ring [147].

#### 3.3.7. TLR9 Agonists

TLR9 recognizes the bacterial DNA motif cytosine-phosphate-guanine (CpG) dinucleotide, activating the immune system through the MyD88 pathway [148]. CpG are molecular motifs that have been modified to prevent protease degradation and used as adjuvants [149]. CpG-CDNs lead to robust chemokine, cytokine and antibody production in natural killer cells, B cells, and pCCS, and evoke a strong Th1-type immune response [150].

CpG1018 is an oligonucleotide able to elicit Th1-type immune response. CpG1018 is one of the four novel adjuvants approved in the last 20 years. Even if its use was initially restricted in Heplisav-B vaccine [151] it is currently under evaluation in vaccines against melanoma and SARS-CoV-2 [152,153].

MGN1703 belongs to the TLR9 agonists and is a small DNA molecule that includes CG motifs and shows a linear structure. It is constituted by double-stranded DNA section in the middle, bordered by two single-stranded structures. It is tested as adjuvant in vaccines against cancers where it activates innate and adaptive immune responses with only mild or temporary side effects [154]. It is currently under evaluation in Phase I and Phase II studies as an immunomodulator alone or in combination for the treatment of malignancies, such as melanoma, small-cell lung cancer, and colorectal carcinoma [155].

### 3.4. CLR Ligands

C-type lectin receptors (CLRs) are immune sensors for lipids derived from pathogens and damaged tissues and are involved in the activation of the innate and acquired immunity [156]. Immune responses can be evoked through CLRs cell signaling pathways and crosstalk with other PRRs, such as TLR, leading to the activation of different signaling pathways and the expression of specific cytokines [157]. The CLR-triggering adjuvants include Curdlan, PGA-45, Trehalose Dibehenate (TDB), and Trehalose dimycolate (TDM), which induce robust Th17 and Th1 responses.

Curdlan is used in *Pseudomonas aeruginosa* vaccine and induces the production of high levels of IL-17A and CD44+ CD62L-CD69+ CD4+ TRM cells [158]. Curdlan is also able to activate dendritic cells (DCs) and enhance DC-based antitumor immunity and for this reason is under evaluation for antitumor immunotherapy [159]. Because of the high hydrophobicity of curdlan, a partially oxidized curdlan derivative β-1,3-polyglucuronic acid, PGA-45 polymer, has been developed. It is able to stimulate phosphorylation of IKK-β and reduce the expression of phosphorylated Akt, suggesting that PGA-45 can activate multiple cell surface receptors, including TLR4 and dectin-1 [160].

Trehalose-6,6′-dimycolate (TDM) and its synthetic analogue trehalose-6,6′-dibehenate (TDB), are able to activate macrophages and dendritic cells through binding to C-type lectin receptor Mincle. TDB is under clinical studies in tuberculosis subunit vaccine. TDB can also act independently of Mincle, inducing the microglial polarization towards M2 phenotype using the PLC-γ1/calcium/CaMKKβ/AMPK pathway, making this adjuvant a therapeutic agent for the treatment of neuroinflammatory diseases [161].

### 3.5. RLR Ligands

Retinoic acid-inducible gene I (RIG-I)-like receptors (RLRs) are sensors of viral infections and induce the production of type I and III interferons and inflammatory cytokines, activating signaling pathways that are involved in innate and acquired immune responses. RLRs can detect both viral and host RNAs, leading to an antiviral response but also to immunopathology if the RLR pathway is uncontrollably activated [147]. Poly(I:C) activates the RLRs pathway since it mimics the viral invasion in cells, leading to the activation of the MAVS-IRF3/7 cascade and the production of IFN-β and ISGs. The expression of IFN-β depends on the length of poly (I:C); a short sequence induces the IFN-β expression in myeloid cells, while a long sequence induces the IFN-β expression in fibroblast cells. This means that the stimulation of RLR pathways with specific agonists will lead to the induction of a cell-specific IFN-β expression, especially in fibroblasts that can confer a stronger antiviral state compared with the monocytes and macrophages [162].

## 4. Co-Adjuvants and Adjuvant Formulation

The majority of vaccines on the market only contain one adjuvant, however frequently the protective immune response is insufficient for vaccine effectiveness. To enhance the effect of vaccines, many adjuvant formulations have been developed. Adjuvants and antigens can be delivered together utilizing one of two popular methods, covalent coupling or packaging the antigen and adjuvant in a delivery mechanism [19]. Proper adjuvant combination choice can result in a complementary or synergistic improvement of the immune response to the vaccine [163]. For example, the use of alum and MPL in combination in AS04 in the HBV and the human papillomavirus (HPV) vaccines show a better immune response in comparison with the same vaccine adjuvanted with just alum salts [7].

### 4.1. Liposomes

Liposomes are spherical particles that can encapsulate antigen and immunostimulatory molecules, protecting them from degradation and delivering them to APCs [164]. Liposomes consist of biodegradable, biocompatible, and nontoxic phospholipids, allowing flexible structure modifications that enable adjustable characteristics, such as size, surface charge, membrane flexibility, and agent loading mode [165]. The use of liposomes as a vaccine adjuvant-delivery system (VADS) has led to many advantages such as high safety and a strong immune response [2]. Liposomal formulations on the market include Epaxal and inflixal administrated against hepatitis A and seasonal influenza virus, respectively [166]. Another liposome-based vaccine is the Shingrix developed by GSK (GlaxoSmithKline) which was approved by FDA for prophylaxis in the elderly of varicella-zoster virus [165].

### 4.2. Emulsions

Emulsions developed as vaccine adjuvants can be divided into two main forms, complete Freund’s adjuvants (CFA) and incomplete Freund’s adjuvants (IFA) [167]. Both these adjuvants are water-in-oil emulsions with the capacity to transport antigens and activate the innate immune system. CFA consists of mineral oil, emulsifier, and heat-killed mycobacteria, which promotes the stimulation of immune responses [2]. However, CFA can cause a strong, long-lasting local inflammation, with the potential development of an ulcer at the injection site [168]. IFA, instead, has the same composition as CFA but it does not contain killed bacteria *M. tuberculosis*. This adjuvant can produce more potent and durable antibody responses compared to the same vaccine without it. However, the use of IFA in vaccine formulation is limited by its strong side effects and toxicity caused by the high levels of non-biodegradable oils and poor quality [169].

#### 4.2.1. Montanide

Montanide is a large family of both oil-in-water and water-in-oil emulsions, and it has been used in both veterinary and human vaccines. The biodegradable nature of the Montanide^TM^ reduces many of the cytotoxic characteristics of IFA, which is similar in physical structure. The formation of a depot at the injection site, which facilitates the antigen’s gradual release, is part of the mechanism of action for this oil-based adjuvant. Concentrated and protected against deterioration antigens are produced, and phagocytosis is promoted [5,170]. Montanide ISA 51 and ISA 720 have been used in several clinical trials involving vaccines for cancer, AIDS, malaria, or autoimmune diseases [171,172]. Montanide ISA 201 and ISA 206, instead, have been used in foot-and-mouth vaccines [173].

#### 4.2.2. MF59

MF59 is a water–oil emulsion adjuvant consisting of squalene, a biodegradable and biocompatible oil that is a normal component of the human body, stabilized in 10 mM sodium citrate buffer at pH 6.5 by the surfactant Tween 80 and Span 85, with an average particle size of less than 200 nm [173]. It was approved for the first time for human vaccine (Fluad) in Europe in 1997 [174], and now it has been administered to more than 100 million people in more than 30 countries. Its mechanism of action is similar to alum salts, with a depot activity at the injection site, that stimulates a local innate immune response [1]. The administration of MF59 in muscle activates powerful cellular and humoral immune response with ATP release and upregulates cytokines and chemokines, which, in turn, promotes leukocyte recruitment, antigen uptake, and migration to lymph nodes to activate B and T cells [53,175,176], resulting in more effective compared to alum salts. MF59 has an acceptable safety profile and is well tolerated, as demonstrated by millions of doses administrated since 1997.

### 4.3. The AS0 Adjuvant Systems

The Adjuvant systems AS0 are based on the combination of the classical adjuvant molecules, such as alum, emulsions, and liposomes. They have been developed by GlaxoSmithKline to achieve the maximum adjuvant effect with acceptable tolerability, combined with immunostimulatory molecules, such as TLR ligands and others [53]. These various combinations of classical adjuvants and immunostimulators have been designed to personalize adaptative immune responses against pathogens in a target population, including young children, elderly, and immunocompromised individuals [6].

#### 4.3.1. AS04

AS04 consists of 3-O-desacyl-4′-monophosphoryl lipid A (MPL), a detoxified form of lipopolysaccharide (LPS), isolated from the Gram-negative bacterium *Salmonella minnesota*, which is adsorbed on alum salts [6]. MPL while being less toxic, retains the immunostimulatory properties of LPS through TLR4 activation [177], when adsorbed on alum. TLR4 signaling on innate cells mediates the adjuvant action of AS04, in association with the intrinsic immunomodulatory properties of alum [178]. We can find AS04 in the hepatitis B virus (HBV) Fendrix [178] and the human papillomavirus (HPV) Cervarix [179] vaccines, showing a better immune response in comparison with the same vaccine adjuvanted with just alum salts [180,181].

#### 4.3.2. AS03

AS03 is a squalene oil-in-water emulsion adjuvant, similar to MF59, combined with the surfactant polysorbate 80 and also α-tocopherol (vitamin E) [182], resulting in less reactogenic with a better safety profile. In 2009, the European Commission approved the commercialization of AS03-adjuvanted vaccine Pandemix, and in 2013 the Food and Drug Administration authorized an AS03-adjuvanted influenza A (H5N1) monovalent vaccine [183]. It is also used in the SARS-CoV-2 recombinant protein vaccine (CoV2 preS dTM) [184]. The antioxidant and immunostimulatory properties of α-tocopherol provoke immune-enhancing response compared to MF59, modulating the expression of certain chemokines and cytokines, such as CCL2, CCL3, IL-6, and GM-CSF [4]. Moreover, AS03 activates the immune system by stimulating NF-κB [185], which causes cytokines and chemokines release in muscles and the draining lymph nodes and promotes the migration of innate immune cells. Additionally, AS03 can promote CD4+ T cell-specific immune responses, which can lead to long-lasting neutralizing antibody production and higher levels of memory B cells [186]. To increase its immunogenicity, the composition of AS03 was further enhanced using two potent immunostimulants, QS-21 (a saponin derived from *Quillaja saponaria*) and 3-O-desacyl-40-monophosphoryl lipid A (MPL), giving rise to AS02 [187,188].

#### 4.3.3. AS01

The AS01 adjuvant is the combination of two potent immunostimulatory components, the TLR4 ligand used also in AS04 (MPL) and an isolated and purified saponin fraction (QS-21), formulated in liposomes. It is used in the shingles vaccine Shingrix and in the malaria vaccine Masquirix [189]. Preclinical studies have shown that QS-21 used as an adjuvant enhances antibody as well as cell-mediated immune responses [190,191] but used as single-component adjuvant in vaccine it has a low tolerability profile. In AS01, the presence of cholesterol in liposome allows it to bind QS-21 and quench its reactogenicity. The MPL activates the innate immune system through TLR4, instead, QS-21 activates caspase 1 in subcapsular sinus macrophages (SSMs) in the draining lymph node [5]. The combination of two well-established adjuvant molecules provokes a synergic activation of innate immunity, that turns out to be greater than the individual sum of the independent components with the activation of novel pathways that are not triggered by either component alone, increasing in polyfunctional CD4+ T cells expressing IL-2, IFNγ, and TNF [192,193]. The use of AS01 as adjuvant is approved in a vaccine against varicella zoster [194], administrated to older adults with high efficacy and in malaria vaccine implemented in a limited campaign in Africa [195].

### 4.4. Immunostimulating Complex

Immunostimulating complexes are another vaccine delivery vehicle with potent adjuvant activity. They are spherical, cage-like self-assembled particles about 40 mm large in size [196,197]. ISCOMs are composed of *Quillaja* saponins, cholesterol, phospholipid, and antigen [198]. The particle without an antigen is known as an ISCOM matrix. The ISCOM antigen can be an envelope protein of a native virus, a cellular membrane protein, or peptides containing hydrophobic domains through apolar interactions [197]. ISCOMATRIX^®^ has been developed using the same material of ISCOMs without the antigen, which can be added during the formulation of the vaccine, enabling more diverse usage and removing the limitation of hydrophobic antigens [170,196,199]. Dendritic cells (DCs) and ISCOM interaction can improve the cross-presentation of the incorporated antigen [200]. Therefore, CD4+ and CD8+ antigen-specific T-cell responses are effectively induced [201]. The induction of cytotoxic T cells, a balanced Th1/Th2 response, and long-lasting antibody responses are all documented effects of ISCOM vaccines [199]. Other ISCOM/ISCOMATRIX vaccines have undergone clinical trials for HIV [202], HPV [6], and cancer utilizing as antigen NY-ESO-1 [6,196]. In all cases, the studies have shown good safety and tolerability profile, as well as induction of humoral and cellular immune responses. While presenting these characteristics, safety concerns regarding the use of ISCOMs in human vaccinations have prevented use because some saponins are toxic at high levels [170].

### 4.5. Virus-Like Particles

Virus-like particles (VLPs) are icosahedral nanoparticles of similar size to viruses (ranging from 20 to 800 nm) [203], which possess the ability to self-assembling capside protein [204]. They are non-infectious particles because they are devoid of genetic material. VLPs are composed of an external viral shell with repetitive epitopes that the immune system recognizes as a non-self and produces a fast and long-lasting immune response, even in the absence of adjuvant [205]. They can be produced by various viral types using diverse technologies and cell systems, such as *Escherichia coli*, yeasts (*Saccharomyces cerevisiae* and *Pichia pastoris*), Baculovirus, mammalian cells, plant cells, or cell-free systems [1]. The historical VLPs manufacturing approach consists of a multisteps methodology called “assemble-then-purify”. The first step consists of the spontaneous assembly of capsid proteins directly inside the expression cell vector. The second step, instead, provides the purification of the newly formed particles. Sometimes it is necessary to disassemble and then reassemble the VLPs in order to obtain well-purified particles and improve quality [206]. Another manufacturing approach for VLPs provides the use of a cell free in vitro assembly, inverting the traditional self-assemble methodology [207,208]. In particular, an in vitro system is used as a platform to induce a spontaneous assembly of capsid proteins after their expression and purification, without the need to disassemble newly formed VLPs [209,210,211]. On the market, two important adjuvanted vaccines use this type of nanoparticle, the hepatitis B (HBV) and papillomavirus (HPV) vaccines. The current hepatitis B vaccine is a recombinant DNA vaccine made utilizing Saccharomyces cerevisiae as the expression vector and includes hepatitis B surface antigen (HBsAg), which is used to prevent hepatitis B infection. This vaccine has been shown to confer immunity for at least 10 years [1,212].

In the HPV vaccine, instead, non-enveloped HPV virions contain double-stranded DNA (dsDNA). The L1 and L2 proteins compose the major and minor structural proteins of the capsid, which has icosahedral symmetry [213]. *Saccharomyces cerevisiae* is the vector currently employed for the expression of L1 proteins. The combination of VLPs and adjuvants (AlP) results in a strong immune response and 90% protection from cervical cancer [214].

### 4.6. Virosome

Virosome is a drug-delivery system that consists of the viral envelope and components of the virus or another pathogen. They are formed by reconstituted influenza virus envelopes and contain hemagglutinin (HA), neuroamidase (NA), and phospholipids and they are lacking viral genetic material, such as VLPs [215]. The first virosome-based vaccine was developed in 1975 and this one is allowed to study the efficacy of this type of vaccine. Since then, two vaccines have reached the market for the prevention of hepatitis A (Epaxal) [216] and influenza (Inflexal) [217].

The presence of HA in the virosome structure allows the maintaining of the receptor-binding capability, increasing the immunogenicity, and membrane fusion activity, but virosomes cannot induce infection in cell after binding because of the lack of viral RNA [218]. Antigens can be transported by virosomes into the cytosol of APCs and induce cytotoxic T lymphocyte responses, making them a perfect delivery system [219]. However, virosomes are not very effective at activating APCs and encouraging cross-presentation because of their poor adjuvant qualities. The use of stronger adjuvants can overcome this inherent constraint. An innovative influenza vaccine, based on virosomes combined with the TLR4 ligand monophosphoryl lipid A (MPLA) and the metal ion-chelating lipid DOGS-NTA, was recently developed [220]. Virosomes can induce strong humoral and cellular immunity, comparable to natural infection and other potent adjuvants. This type of delivery system has been approved by FDA for human use due to their very high tolerance and safety profile [221,222]. In addition to the two virosome-based vaccines against influenza and hepatitis A, several vaccines are under clinical trials, including those against HIV [223], HPV [224], RSV [218], and malaria.

## 5. New Insights into Adjuvants Design and Development

As listed before, novel classes of immune-activating adjuvants have been discovered in the last 20 years, able to boost immune response in fragile populations such as immunocompromised and elderly people. Several classes of adjuvants are available, each of them with its own strengths and weaknesses. Delivery systems such as oil-in-water emulsions are effective and safe systems, nevertheless, suffer from the possibility to create local or systemic adverse effects. Recent studies demonstrate that PRR signaling can be altered in several populations, reducing the protective effect of vaccine formulation. As an example, the immunological mechanisms that prevent fetal rejection as well as the dysregulation of TLR pathway in elderly people or the presence of genetic polymorphisms [225]. For all these reasons, the choice of the proper adjuvant formulation is of primary importance to increase efficacy without reducing the risk–benefit ratio. From the chemical point of view, several crystallographic structures of proteins co-crystalized with specific ligands are available on the protein data bank repository [226], facilitating the search for novel compounds through in silico-aided drug design. Most of the structures reported for STING and TLR small molecule agonists are heterocyclic compounds, mimic of the nucleobases. Even in this case, many structurally related compounds have been already developed with different applications, including kinase inhibitors with broad applications in cancer disorders and poorly known mechanisms of action [227] or ligands of human cofactors [228,229,230]. The existence of high-throughput fully automated instruments for the synthesis of peptides is another aspect that should be taken into consideration. Recent efforts have been made to synthesize complex high molecular-weight peptides and glycoproteins [231]. In addition, novel complex nanoformulations have been developed, using natural components, such as functionalized polymers derived from insects. All these aspects are extremely promising and open novel scenarios in the development of adjuvant discovery.

## 6. Conclusions

Adjuvants are a large family of key components of vaccine formulations. Their use has been fundamental during the most important vaccination campaigns of history, including Polio, swine flu, and the last COVID-19 pandemic. Even if good results have been already obtained, other efforts are fundamental to increase vaccine protection against resistant viruses and reduce the necessity of additional booster shots. Recombinant technology, DNA screening, and bioinformatic research have clarified novel mechanisms of action and key players of the immune signaling pathways. Other efforts are necessary to identify more potent adjuvants able to counteract future pandemics and increase the chance of success against cancer.

## Figures and Tables

**Table 1:** Classification of novel delivery systems and immune potentiators adjuvants.

	Classification		Adjuvants
Delivery Systems	Aluminium salts		AS04, Alum + CpG
	Emulsions	O/W	MF59, AS02, AS03, AF03, MPL-SE, GLA-SE, SLA-SE
		W/O	Montanide ISA-720, Montanide ISA-51
	Nanoparticles	Liposomes	AS01, AS015
		Lipid-membrane based	Virosomes, Archaeosomes
Immune Potentiators	TLR Agonists	TLR2	L-pampo, MALP-2, PAM_2_CSK_4_, PAM_3_CSK_4_, lipoarabinomannans, lipoteichoic acids, GP1 anchors, zymosan, peptidoglican
		TLR3	Poly(I:C) (polyinosininc:polycytidylic acid) Poly-ICLC, ARNAX
		TLR4	AS0, Monophosphoryl lipid A (MPL)
		TLR5	Flagellin, Imiquimod (R837), Resiquimod (R848)
		TLR7/8	Imiquimod and Resiquimod
		TLR9	CpG-B-ODN, CpG1018, MGN1703

**Table 2:** Aluminum adjuvant containing vaccines approved by FDA and type of adjuvant.

Vaccine	Trade Name	Manufacturer	Adjuvant
Anthrax	BioThrax	Emergent BioSolutions	AH
Diphtheria, tetanus toxoids adsorbed	None	Sanofi Pasteur	AP
	TDVAX	MassBiologics	AP
Tetanus and Diphtheria toxoids, adsorbed	Tenivac	Sanofi Pasteur	AP
	None	Sanofi Pasteur	AP
DTaP	Infanrix	GSK	AH
	Daptacel	Sanofi Pasteur	AP
TdaP	Adacel	Sanofi Pasteur	AP
	Boostrix	GSK	AH
DTaP, Polio	Kinrix	GSK	AH
	Quadracel	Sanofi Pasteur	AP
DTaP, Polio, Hep B, Hib	Vaxelis	MSP Vaccine Company	AP
DTaP, Polio, Hib	Pentacel	Sanofi Pasteur	AP
Hib	PedvaxHIB	Merck	AAHS
Hep A	Havrix	GSK	AH
	VAQTA	Merck Sharp & Dohme	AAHS
Hep A, Hep B	Twinrix	GSK	AH; AP
Hep B	Recombivax HB	Merck	AAHS
	Prehevbrio	VB1 Vaccines	AH
	Engerix-B	GSK	AH
HPV	Gardasil	Merck	AAHS
	Gardasil 9	Merck	AAHS
	Cervarix	GSK	AH
JEV	Ixiaro	Valneva Austria	AH
Meningococcus B	Bexsero	GSK	AH
Pneumococcus	Prevnar 13	Pfizer	AP
	Vaxneuvance	Merck Sharp & Dohme	AP
	Prevnar 20	Pfizer	AP
TBE	Ticovac	Pfizer	AH

AH: aluminum hydroxide; AP: aluminum phosphate; AHHS: aluminum hydroxyphosphate sulphate; DTaP: diphtheria toxoid, tetanus toxoid, acellular pertussis; TdaP: tetanus toxoid, reduced diphtheria toxoid, acellular pertussis; Polio: poliomyelitis; Hib: Hemophilus Influenzae B; Hep A: Hepatitis A; Hep B: Hepatitis B; HPV: Human Papillomavirus; JEV: Japanese Encephalitis Virus; TBE: tick-borne encephalitis. Information accessed on FDA website on 29 November 2022 (https://www.fda.gov/vaccines-blood-biologics/vaccines/vaccines-licensed-use-united-states) [28,43].

**Table 3:** STING agonists in clinical trials.

Agonist Class	Agonist	Status	Indications	Therapy	NCT Code ^a^
CDN	CDK 002	Phase I/II	Advanced/metastatic solid tumors	Single	NCT04592484
	MK-2118	Phase I	Advanced/metastatic solid tumors, lymphoma	Single or combination	NCT03249792
	SB-11285	Phase I	Advanced solid tumors, melanoma	Single or combination	NCT04096638
	IMSA-101	Phase I/II	Advanced solid tumors	Single or combination	NCT04020185
	TAK 676	Phase I	Advanced or metastatic solid tumors Carcinoma; NSCLC, Triple Negative Breast Neoplasms, HNSCC	Single or combination Combination	NCT04420884 NCT04879849
	SYNB1891	Phase I	Advanced solid tumors, lymphoma	Single or combination	NCT04167137
	BI1387446	Phase I	Advanced solid tumors	Single or combination	NCT04147234
NCDN	BMS-986301	Phase I	Advanced solid tumors	Single or combination	NCT03956680
	GSK3745417	Phase I Phase I	Advanced solid tumors Relapsed or Refractory Myeloid Malignancies Including Acute Myeloid Leukemia (AML) and High-risk Myelodysplastic Syndrome (HR-MDS)	Single or combination	NCT03843359 NCT05424380
	SNX281	Phase I	Advanced solid tumors Advanced Lymphoma	Single or combination	NCT04609579

CDN: cyclic dinucleotides; NCDN: non-cyclic dinucleotides small molecules; HNSCC head and neck squamous cell carcinoma; NSCLC: non-small cell lung carcinoma. ^a^ NCT code: unique identification code given to each clinical study on www.clinicaltrials.gov (accessed on 20 December 2022).

**Table 4:** Pathogen-associated molecular patterns (PAMPs) recognized by Toll-like receptors.

PAMPs	Toll-like Receptors
Lipo-polysaccharides	TLR4
Lipopeptides	TLR2 + TLR6 or TLR1
Single-stranded RNA	TLR7/8
Double-stranded RNA	TLR3
CpG motif containing DNA	TLR9

**Table 5:** Toll-like receptors agonists in clinical trials.

Agonist Class	Agonist	Status	Indications	Therapy	NCT Code ^a^
TLR-2	Poly-ICLC	Phase I Phase I Phase I/II Phase II	Malignant Pleural Mesothelioma COVID-19 Non-Hodgkin’s Lymphoma, Metastatic Breast Cancer, Head and Neck Squamous Cell Carcinoma Low-grade Glioma	In combination	NCT04525859 NCT04672291 NCT03789097 NCT02358187
TLR-4	GLA-SE	Phase I Phase I Phase I	Schistosomiasis HIV Infections Malaria	In combination	NCT05292391 NCT04607408 NCT05644067
	AS04	Phase I	HIV infections	Single	NCT04301154
	TAK-242	Phase II	Acute Alcoholic Hepatitis	Single	NCT04620148
TLR-7	Imiquimod	Phase III Phase III Phase III Phase I Phase I	Anogenital Human Papillomavirus Infection Condyloma Anal Influenza Carcinoma, Squamous Cell Cervical Cancer Carcinoma, Squamous Cell	Single or combination	NCT03289260 NCT04143451 NCT04143451 NCT00788164 NCT03370406
TLR 7/8	M5049	Phase I Phase II Phase II Phase II	Systemic and cutaneous Lupus Erythematosus Dermatomyositis and Polymyositis Systemic Lupus Erythematosus Systemic Lupus Erythematosus	Single or combination	NCT04647708 NCT05650567 NCT05162586 NCT05540327
TLR-9	IMO-2125	Phase II	Malignant Melanoma	In combination	NCT04126876
	SD-101	Phase I Phase I/II	Metastatic Uveal Melanoma in the Liver Hepatocellular Carcinoma, Intrahepatic Cholangiocarcinoma	In combination	NCT04935229 NCT05220722

^a^ NCT code: unique identification code given to each clinical study on www.clinicaltrials.gov (accessed on 23 December 2022).

## Data Availability

Not applicable.
